# Hydroxyurea-Induced Acute Pancreatitis

**DOI:** 10.7759/cureus.26132

**Published:** 2022-06-20

**Authors:** Harjot Bath, Khushmanjit Jawandha, Mohammed G Elhassan

**Affiliations:** 1 Internal Medicine, Saint Agnes Medical Center, Fresno, USA; 2 Internal Medicine, California Health Sciences University, Fresno, USA

**Keywords:** jak 2 mutation, adult gastroenterology, hydroxyurea, drug induced pancreatitis, adverse drug event, essential thrombocythemia treatment, acute pancreatitis (ap)

## Abstract

Drug-induced pancreatitis (DIP), while not a major cause of acute pancreatitis, remains a debilitating diagnosis resulting in significant patient morbidity and mortality. The diagnosis includes first diagnosing acute pancreatitis, second ruling out more common etiologies (alcohol abuse, gallstones, etc.), and third documenting a thorough history (in particular medications). Essentially, it is a diagnosis of exclusion. Any drugs with the potential to result in acute pancreatitis should be discontinued, and those without future recurrence of pancreatitis are deemed to have had a drug-induced case. Although the exact pathophysiology of the initial development of DIP is unknown, we hypothesize it is different for various drug classes. It is known that once pancreatic enzymes are activated after insult, they activate an inflammatory response resulting in auto-digestion of the pancreas. Our report discusses a previously not documented case of DIP in a patient on hydroxyurea monotherapy for the treatment of Janus kinase 2 (JAK2) essential thrombocytosis.

## Introduction

Each year in the US, well over 100,000 hospital admissions occur due to acute pancreatitis, with drug-induced pancreatitis (DIP) accounting for a minority of these cases (less than 3%) [1]. Over the last half-century, a steady rise in the incidence of acute pancreatitis in Western countries has been identified [2]. It remains imperative to recognize acute pancreatitis as it contributes to the economic burden on healthcare due to increased hospital readmission and excessive work-up cost. Diagnosis of DIP includes ruling out common causes of pancreatitis, including gallstones, ethanol abuse, hypercalcemia, hypertriglyceridemia, and trauma [3]. Thereafter early identification of inciting drug allows for a shorter hospital stay, decreased risk of complications associated with prolonged hospital admission, and secondary prevention of repeat pancreatitis [1].

A plethora of new medications are regularly being introduced to the pharmaceutical market and administered by clinicians, resulting in an increased number of drugs that could potentially be implicated in DIP. The source of the available data in DIP originates from case series, case reports, or case-control studies [2]. This is a case report of acute pancreatitis attributed to the use of hydroxyurea, a medication not well known to be associated with pancreatitis.

## Case presentation

An 80-year-old female with a past medical history of Janus kinase 2 (JAK2) polycythemia vera, hypertension, transient ischemic attack presented to the emergency department with a one-day history of increasing, constant epigastric abdominal pain, which radiated to her back. The nature of the pain was described as achy, and the severity was nine out of ten. She denied any apparent trigger or alleviating factor. Associated symptoms included nausea and vomiting (non-bilious, non-bloody). She had denied fever, diarrhea, chest pain, melena, or hematochezia. 

Admission medications included hydroxyurea 1500 mg daily, amlodipine 5 mg daily, atorvastatin 40 daily, and hydrochlorothiazide (HCTZ) 12.5 mg - lisinopril 20 mg combination pill daily. The atorvastatin was started 18 months prior to DIP admission, amlodipine 16 months prior, and HCTZ-lisinopril 15 months prior. She was closely followed in an outpatient clinic within a week of being started on atorvastatin until after DIP admission, and no side effects were reported at any of her several visits. Of note, none of the medications, except hydroxyurea, were altered in dosage in the year leading up to admission. She had been started on hydroxyurea 17 weeks prior to admission on 500 mg twice daily dosing. Of note, approximately 4.5 weeks prior to admission, her hydroxyurea dose was increased to three times daily dosing. There was no use of over-the-counter medications, no short-term medications (i.e., antibiotics), or herbal substances. No known drug allergies. No history of recent surgery or abdominal surgery/procedures. 

In regards to her social history, the patient was a retired respiratory therapist. Her alcohol intake at the time of admission included one beer once or twice a month at most, which is unchanged from before as she has never been a heavy drinker. She denied a history of illicit drug use or cigarette/marijuana smoking. Her vital signs on admission were a temperature of 36.6 Celsius, heart rate of 72 beats per minute, oxygen saturation of 97% on ambient air, and blood pressure was 173/100 mmHg. 

A physical exam revealed an elderly female in mild distress due to pain. She had a soft, non-distended abdomen which was tender to palpation in the epigastric, left upper quadrant, and left lower quadrant.

**Figure 1:**
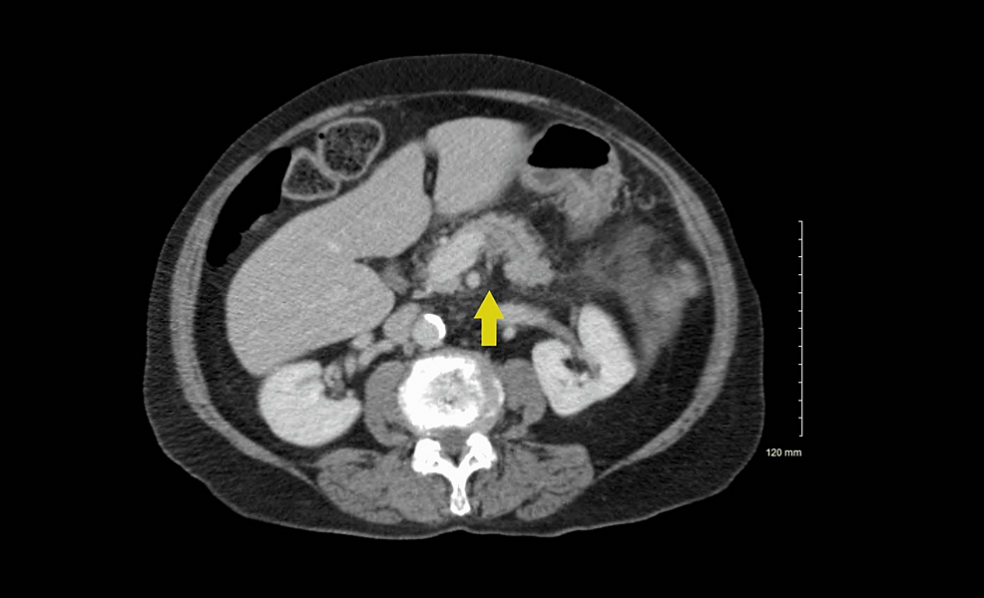
CT of abdomen pelvis with contrast Arrow is demonstrating pancreatitis.

Imaging on the morning admission included limited abdomen ultrasound, which revealed normal liver echogenicity without any focal lesion or surface nodularity. The pancreas appearance was not documented in the right upper quadrant ultrasound report. There was no sonographic evidence of acute cholecystitis or gallstones, ruling out gallstone pancreatitis. Alcohol abuse was deemed less likely as there was no cirrhosis/steatohepatitis present, a low degree of alcohol intake, and no laboratory evidence of long-term alcohol use. There was no alcohol level noted, and her son, who is highly involved in care, corroborated no recent alcohol use. One hour later, a CT scan with contrast reported faint stranding and fluid centered around the pancreas consistent with acute interstitial edematous pancreatitis (Figure 1). 

**Figure 2:**
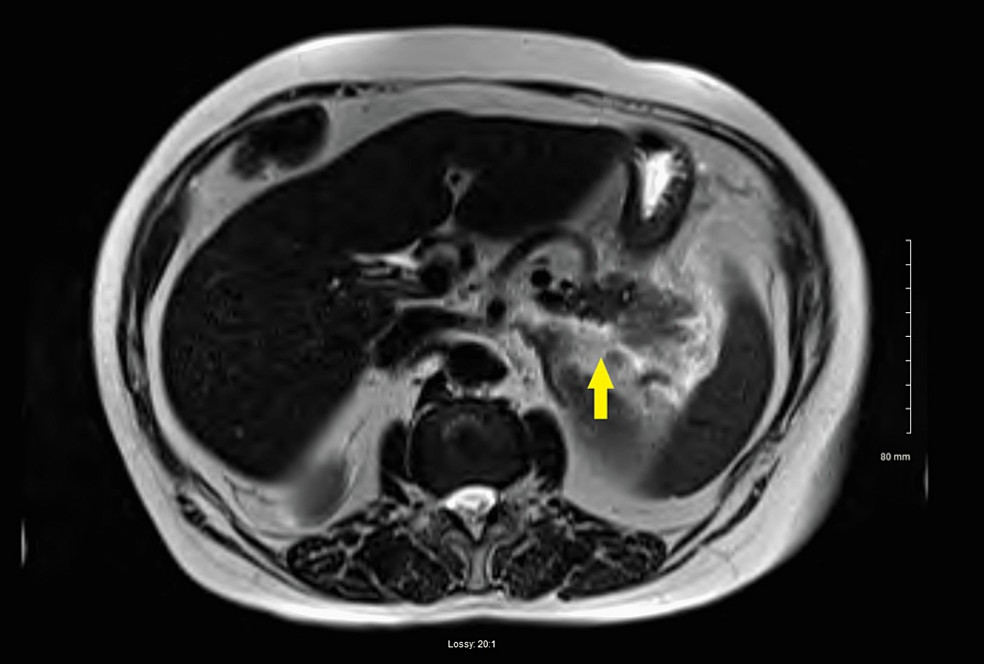
MRI of the abdomen with contrast Arrow is demonstrating pancreatitis.

Later in the afternoon on the day of admission, magnetic resonance imaging of the abdomen with and without contrast reported mild-moderate pancreatic fluid compatible with acute pancreatitis expected on prior CT (Figure 2). There was no evidence of intrahepatic/extrahepatic bile duct dilation, cholecystitis, or gallstones. The common bile duct measured 4 mm, making gallstone pancreatitis unlikely.

Standard work-up to determine acute pancreatitis etiology ruled out alcohol-related pancreatitis as the patient only consumed one or two beers a month at most, if even that. Relevant lab studies are summarized in Table 1. To rule out hypertriglyceridemia, a lipid panel was ordered and was within normal limits. Hypercalcemia was also ruled out as the level was 8.6 mg/dL (normal range 8.5 to 10.5). Autoimmune pancreatitis work-up was negative, which included IgG1-4 subclasses. 

**Table 1:** Admission laboratory results

Serum test	Result (normal range)
Sodium	133 mMol/L (135-145)
Potassium	3.4 mMol/L (3.5-5.5)
Chloride	99 mMol/L (98-114)
Carbon dioxide level	28 mMol/L (21-31)
Creatinine	0.71 mg/dL (0.5-1.2)
Calcium	8.6 mg/dL (8.5-10.5)
Triglycerides	40 mg/dL (<200 )
Alkaline phosphatase	54 Units/L (18-125)
Alanine transaminase (ALT)	42 Units/L (4-23)
Aspartate transaminase (AST)	35 Units/L (9-35)
Mean corpuscular value (MCV)	92.8 nml (81-99)
Total / direct bilirubin	0.8 (0.1-1.3) / 0.2 mg/dL (0.0-0.2)
Amylase	2,520 Units/L (29-103)
Lipase	5,880 Units/L (22-151)
Hemoglobin	12.9 gm/dL (12-16)
White blood cell count	8 thou/mcL (4.5-11)

Along with suspending all home medications (hydroxyurea, amlodipine, atorvastatin, and HCTZ-lisinopril) due to intolerance of oral medications, the patient received supportive treatment with antiemetic agents such as metoclopramide, ondansetron, fluid resuscitation and pain control with hydromorphone. She also had no noted hypotensive episodes during hospitalization. Her outpatient hematologist was consulted and attributed pancreatitis to hydroxyurea. Her baseline platelets prior to any treatment were in the 800s and had been reduced with hydroxyurea to normal levels, also seen on admission. All of her initial symptoms improved upon discharge, and she was continued on all aforementioned home medications except hydroxyurea.

Her JAK2 polycythemia vera treatment was switched from hydroxyurea to ruxolitinib 25 mg twice daily, which she has tolerated. She denied any further abdominal pain, nausea, vomiting, diarrhea, or constipation during follow-up clinic visits eight months post-admission.

## Discussion

Hydroxyurea has many uses, including but not limited to lowering platelet levels in thrombocytosis, treatment in sickle cell, and as a chemotherapy agent. It exhibits cytotoxic effects on cells and prevents de novo DNA synthesis and DNA repair. It accomplishes this by inhibiting ribonucleotide reductase in the DNA synthesis pathway, specifically blocking the S phase of the cell cycle [4,5]. During the hydroxyurea-ribonucleotide reductase interaction, hydroxyurea will bind to and block the ribonucleotide reductase active site [6]. Ultimately, this leads to cell death. 

This patient's history of JAK2 essential thrombocytosis predisposes her to thrombosis formation, which is a significant cause of morbidity and mortality in this patient population and is reported to occur in 20% of patients [7]. There was no occluded pancreatic vessel or necrosis seen on imaging. It could have been a transient occlusion; however, if that was the case, the occurrence of ischemia-implicated pancreatitis is less likely. Pancreatic duct abnormalities causing pancreatitis were ruled out by imaging. Splenic vein, superior mesenteric vein, and portal vein thrombosis are typical complications of acute pancreatitis, not documented to be inciting factors [8]. It can be argued that statins, hydrochlorothiazide, amlodipine or even lisinopril [9] can induce pancreatitis. However, in our case, they were ruled out as the patient was continued on these medications on discharge without side effects during the eight months of close follow-up since discharge. 

Our case is unique as there have not been any documented case reports of DIP with the use of hydroxyurea monotherapy for JAK2 polycythemia vera. The cases of DIP attributed to hydroxyurea are reported to have occurred in HIV-positive patients on concurrent highly active antiretroviral therapy (HAART) drugs [6]. Nucleoside reverse transcriptase inhibitors (NRTIs) are implicated in the onset of acute pancreatitis, with their therapeutic effect meant to be intensified by hydroxyurea [10]. A DIP classification system proposed by Badalov et al. classifies implicated medications into four classes. This system of DIP classification groups drugs into one of four classes based on the number of case reports, positive re-challenge results, an established latency period, and whether or not alternative causes of acute pancreatitis were ruled out [11]. In our patient, all medications she was on during admission were "re-challenged" and did not cause pancreatitis and therefore ruling them out as the inciting drug. Class 4 in the classification refers to a drug with a single case report without a re-challenge test, such as hydroxyurea. Although there are causative agent classification systems that exist, it is important to routinely amend and revise these systems so that they can be kept up to date. Moreover, less commonly prescribed and over-the-counter medications are oftentimes not included in these classification systems allowing numerous cases to go unreported.

## Conclusions

In conclusion, the benefits of reporting and identifying which drugs contribute to DIP include a decrease in hospitalizations, patient morbidity/mortality, and hospital readmission, as well as a shorter length of stay in hospital; all of which would also lower the economic impact that DIP has on the healthcare system. It is also important to note that many reported cases of DIP do not have a re-challenge test or a drug latency period that can definitively associate acute pancreatitis with a specific medication due to ethical concerns. This further contributes to the difficulty in determining an accurate diagnosis of DIP. In our patient case, completing a thorough work-up to rule out more common causes of acute pancreatitis, documenting a complete medication list, along with permanently discontinuing hydroxyurea have been successful in preventing further episodes of drug-induced acute pancreatitis.
